# PIWI-Like 1 and PIWI-Like 2 Expression in Breast Cancer

**DOI:** 10.3390/cancers12102742

**Published:** 2020-09-24

**Authors:** Ramona Erber, Julia Meyer, Helge Taubert, Peter A. Fasching, Sven Wach, Lothar Häberle, Paul Gaß, Rüdiger Schulz-Wendtland, Laura Landgraf, Sabrina Olbricht, Rudolf Jung, Matthias W. Beckmann, Arndt Hartmann, Matthias Ruebner

**Affiliations:** 1 Institute of Pathology, Comprehensive Cancer Center Erlangen—Europäische Metropolregion Nürnberg(EMN), University Hospital Erlangen, Friedrich-Alexander University Erlangen-Nürnberg (FAU), 91054 Erlangen, Germany; laura.landgraf@fau.de (L.L.); sabrina.olbricht@fau.de (S.O.); rudolf.jung@uk-erlangen.de (R.J.); arndt.hartmann@uk-erlangen.de (A.H.); 2 Department of Gynecology and Obstetrics, Comprehensive Cancer Center Erlangen-EMN, University Hospital Erlangen, Friedrich-Alexander-University Erlangen-Nuremberg (FAU), 91054 Erlangen, Germany; julia.meyer@uk-erlangen.de (J.M.); peter.fasching@uk-erlangen.de (P.A.F.); lothar.haeberle@uk-erlangen.de (L.H.); paul.gass@uk-erlangen.de (P.G.); matthias.beckmann@uk-erlangen.de (M.W.B.); 3 Department of Gynecology and Obstetrics, Biostatistics Unit, Erlangen University Hospital, Friedrich-Alexander-University of Erlangen-Nuremberg (FAU), 91054 Erlangen, Germany; 4 Department of Urology and Pediatric Urology, Comprehensive Cancer Center Erlangen-EMN, University Hospital Erlangen, Friedrich-Alexander University Erlangen-Nuremberg (FAU), 91054 Erlangen, Germany; helge.taubert@uk-erlangen.de (H.T.); sven.wach@uk-erlangen.de (S.W.); 5 Institute of Diagnostic Radiology, Comprehensive Cancer Center Erlangen-EMN, University Hospital Erlangen, Friedrich-Alexander-University Erlangen-Nuremberg (FAU), 91054 Erlangen, Germany; ruediger.schulz-wendtland@uk-erlangen.de

**Keywords:** PIWI-like 1, PIWI-like 2, breast cancer, Luminal A, Luminal B, HER2, TNBC, PIWIL1, PIWIL2, molecular breast cancer subtypes

## Abstract

**Simple Summary:**

A family of proteins, the PIWI proteins, play a crucial role in the regulation of the development of germ cells and self-preservation of so-called stem cells. Former studies have shown that these proteins can be over- or underrepresented (over-/underexpressed) in some cancers and, in the case of abnormal expression, may be correlated with worse outcomes of tumor patients. In our study, we investigated the influence of the two PIWI proteins, PIWI-like 1 and PIWI-like 2, on the survival of breast cancer patients and their correlation with certain breast cancer subtypes. If a breast cancer showed a higher expression of PIWI-like 1 protein but less PIWI-like 2 protein than in non-tumorous tissue, the patient suffered from a more aggressive breast cancer subtype and had shorter survival. By analyzing these two proteins in breast cancer, we were able to predict tumor aggressiveness and prognosis.

**Abstract:**

PIWI-like 1 and PIWI-like 2 play a role in stem cell self-renewal, and enhanced expression has been reported for several tumor entities. However, few studies have investigated PIWI-like 1 and PIWI-like 2 expressions in breast cancer subtypes regarding prognosis. Therefore, we examined protein expression in a large consecutive cohort of breast cancer patients and correlated it to breast cancer subtypes and survival outcome. PIWI-like 1 and PIWI-like 2 expressions were evaluated using immunohistochemistry in a cohort of 894 breast cancer patients, of whom 363 were eligible for further analysis. Percentage and intensity of stained tumor cells were analyzed and an immunoreactive score (IRS) was calculated. The interaction of PIWI-like 1 and PIWI-like 2 showed a prognostic effect on survival. For the combination of high PIWI-like 1 and low PIWI-like 2 expressions, adjusted hazard ratios (HRs) were significantly higher with regard to overall survival (OS) (HR 2.92; 95% confidence interval (CI) 1.24, 6.90), disease-free survival (DFS) (HR 3.27; 95% CI 1.48, 7.20), and distant disease-free survival (DDFS) (HR 7.64; 95% CI 2.35, 24.82). Both proteins were significantly associated with molecular-like and PAM50 subgroups. Combining high PIWI-like 1 and low PIWI-like 2 expressions predicted poorer prognosis and both markers were associated with aggressive molecular subtypes.

## 1. Introduction

*P*-element-induced wimpy testis (PIWI) proteins belong to the Argonaute protein family, which contains the eIF2C/AGO and the PIWI subfamily. The four genes found in the human genome, *PIWIL1, PIWIL2, PIWIL3*, and *PIWIL4*, encode for the proteins PIWI-like 1, PIWI-like 2, PIWI-like 3, and PIWI-like 4, respectively [1]. In general, PIWI proteins are highly conserved in fungi, plants, animals, and humans; contain PAZ and PIWI motifs [2]; and were first reported in *Drosophila melanogaster* [3,4]. Gene and protein (over)expressions are predominantly found in germline cells of the testis and ovary, respectively, and hematopoietic stem cells. By forming complexes with PIWI-interacting RNA (piRNA), a class of non-coding small RNAs, PIWI proteins play crucial roles in genetic and epigenetic regulation of germline gene expression, gametogenesis, and stem cell self-renewal. They harbor an endoribonuclease function and are involved in RNA silencing, transposon regulation chromatin remodeling, and cell differentiation [2,5,6,7,8].

Aberrant expression of piRNA and PIWI proteins has been identified in various types of tumor cells, including seminoma of the testis, sarcomas, and carcinomas [2,9,10,11,12]. In colorectal carcinoma and hepatocellular cancer, immunohistochemical expression of PIWI-like 1 has been associated with poor outcomes. Positivity for PIWI-like 1 predicted chemo-resistance in cervical cancer patients [2,13,14,15]. PIWI-like 2 has been reported to be associated with neoplastic colon tissue [16] and poor outcome [17] in colon carcinoma. It acts as an inhibitor of apoptosis and promotes proliferation via STAT3/Bcl-XL and STAT3/Cyclin D1 pathways [18]. In somatic breast tissue, 676 individual piRNAs have been described with lengths ranging between 26 and 32 nt with 25 piRNAs being differentially expressed in breast cancer. Using piRNA expression for risk calculation, breast cancer patients may be divided into prognostic relevant risk groups regarding survival [19]. Comparing nonneoplastic breast cell lines with breast cancer cell lines, piRNA expression pattern differed between normal and cancer cell lines [7]. Both PIWIL1 mRNA and PIWI-like 1 protein overexpressions are reported in breast cancer. In the breast cancer cell lines MDA-MB231 and MCF-7, overexpression correlated with cell growth promotion and is associated with tumor size, grading, and positive lymph nodes [20]. In one breast cancer study, PIWI-like 1 and PIWI-like 2 showed higher expression levels in invasive ductal carcinoma when compared to mastopathy tissue. Both markers are correlated with each other. PIWIL1 mRNA has not be found in normal breast epithelium. However, PIWIL2 has shown higher expression in nonneoplastic breast tissue than in breast cancer [21]. Few studies have analyzed the immunohistochemical (IHC) expression of PIWI-like 1 and PIWI-like 2, respectively, in breast cancer subtypes and their influence on survival. Hence, we investigated the expression of these proteins in breast cancer, their relation to subtypes and studied their influence on outcome in breast cancer patients.

## 2. Results

### 2.1. Patients

**Figure 1:**
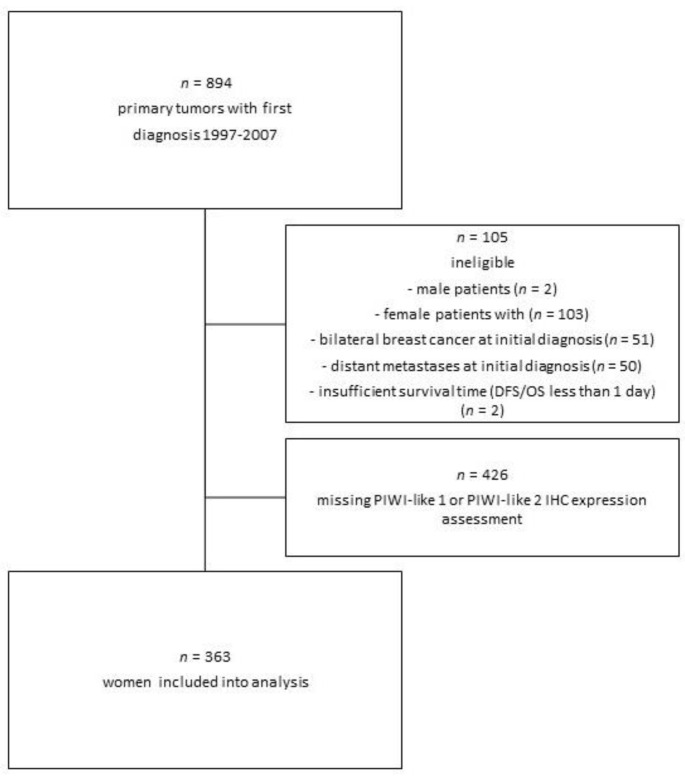
Patient selection and exclusion criteria. Abbreviations: IHC—immunohistochemistry; DFS—disease-free-survival; OS—overall survival.

The final dataset contained 363 patients with complete information on PIWI-like 1 and PIWI-like 2 as well as tumor characteristics and survival data (Figure 1). The median age of the patients was 58 years, with an interquartile range (IQR) of 49–67 years. Tumors were mostly hormone receptor-positive, of intermediate grade, and mostly of Luminal like subtype. Baseline clinical and pathologic characteristics are shown in Table 1.

### 2.2. Distribution of PIWI-Like 1 and PIWI-Like 2

**Figure 2:**
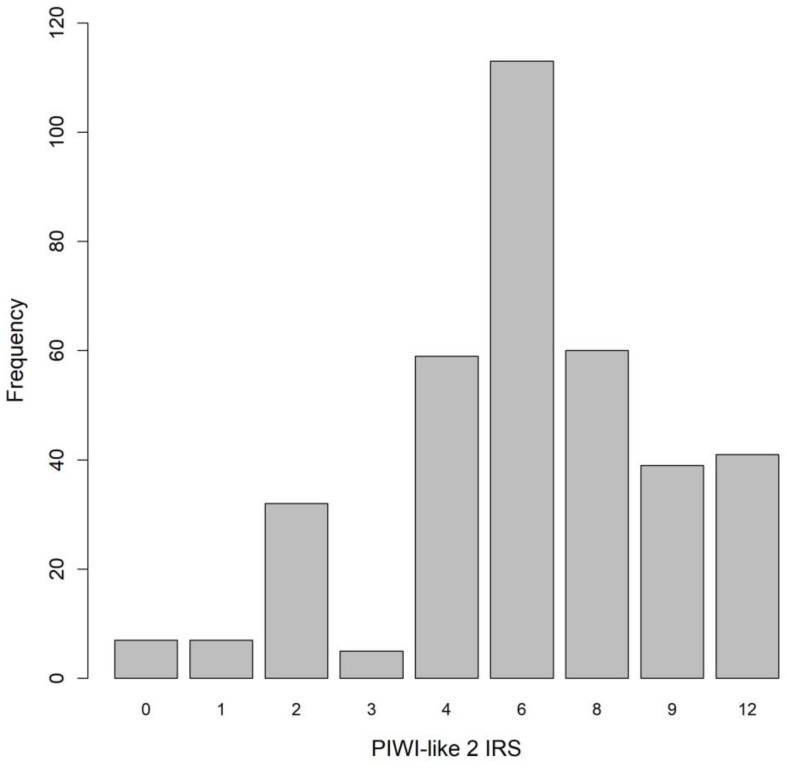
Distribution of PIWI-like 2 IRS in invasive breast cancer.

**Figure 3:**
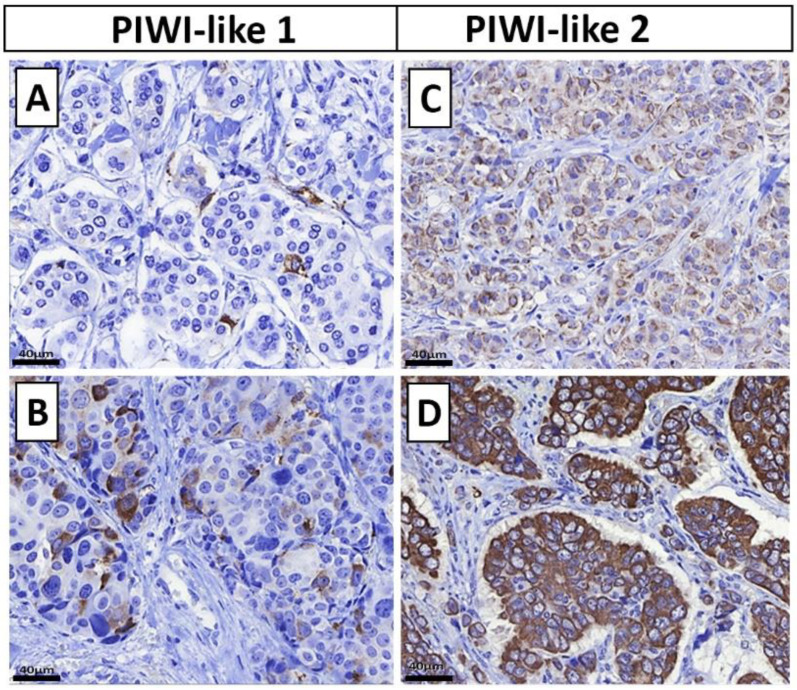
(**A**,**B**) Immunohistochemical staining of PIWI-like 1 and (**C**,**D**) PIWI-like 2 in breast cancer (IHC, 400×). In general, most tumors showed no or only slight PIWI-like 1 expression; few tumors showed a higher PIWI-like 1 staining: PIWI-like 1 IHC expression is almost negative in (**A**), whereas it is easily recognizable in (**B**). Note the cytoplasmic staining. Although most breast cancer cases showed PIWI-like 2 expression, there were differences in intensity and percentage of positively-stained tumor cells identifiable: PIWI-like 2 membranous and cytoplasmic staining is weak in (**C**), but strong in (**D**).

In general, we found mostly cytoplasmic staining for PIWI-like 1, with only a few cases expressing it sparsely in the nucleus. Therefore, nuclear staining was not assessed for further analysis. For PIWI-like 2, there was cytoplasmic and membranous staining, but no nuclear staining was observed. The PIWI-like 1 immunoreactive score (IRS) was greater than zero in 12.9% (*n* = 47, median IRS = 0, IQR: 0–0, Table 1). For PIWI-like 2, the median IRS was 6 (IQR: 4–8) and IRS ≥ 6 was found in 69.7% of patients (Table 1). The distribution of PIWI-like 2 IRS is depicted in Figure 2. Immunohistochemical staining of PIWI-like 1 and PIWI-like 2 is illustrated in Figure 3, showing the cell compartments stained as well as varying intensity and percentage of the protein expression.

### 2.3. Overall Survival

**Figure 4:**
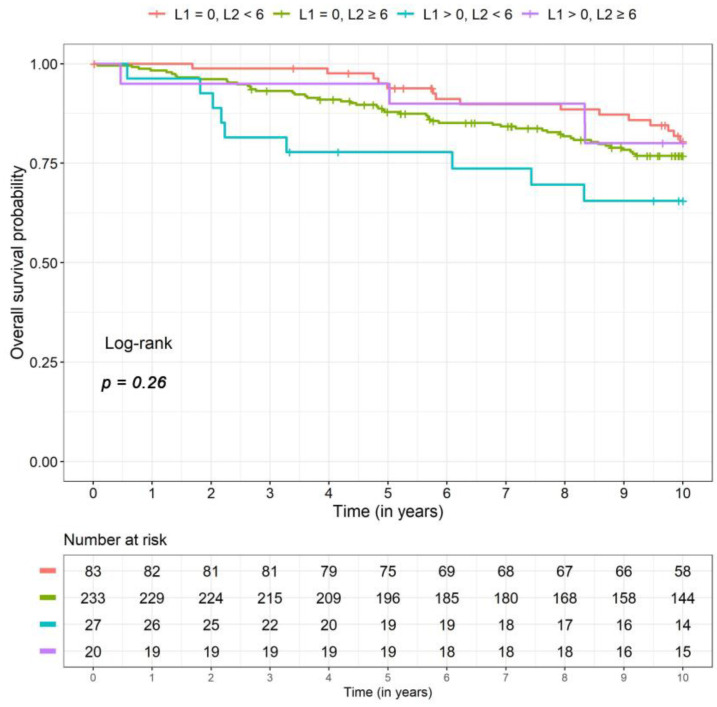
Kaplan–Meier curves for the four different combinations of PIWI-like 1 IRS (L1) and PIWI-like 2 IRS (L2) for overall survival.

The interaction of PIWI-like 1 and PIWI-like 2 had a significant prognostic effect on overall survival *(p =* 0.03). Table 2 presents hazard ratios (HRs) for the four different combinations of PIWI-like 1 and PIWI-like 2. Patients with high PIWI-like 1 IRS (>0) and low PIWI-like 2 IRS (<6) showed a 2.9-fold risk (adjusted HR) compared to patients with both values low (PIWI-like 1 IRS = 0, PIWI-like 2 IRS < 6). This patient group exhibited the lowest 5- and 10-year survival rates (Table 3). Note, that there were only nine events in that group. Kaplan–Meier curves for the four combinations of PIWI-like 1 and PIWI-like 2 are illustrated in Figure 4.

### 2.4. Disease-Free and Distant Disease-Free Survival

**Figure 5:**
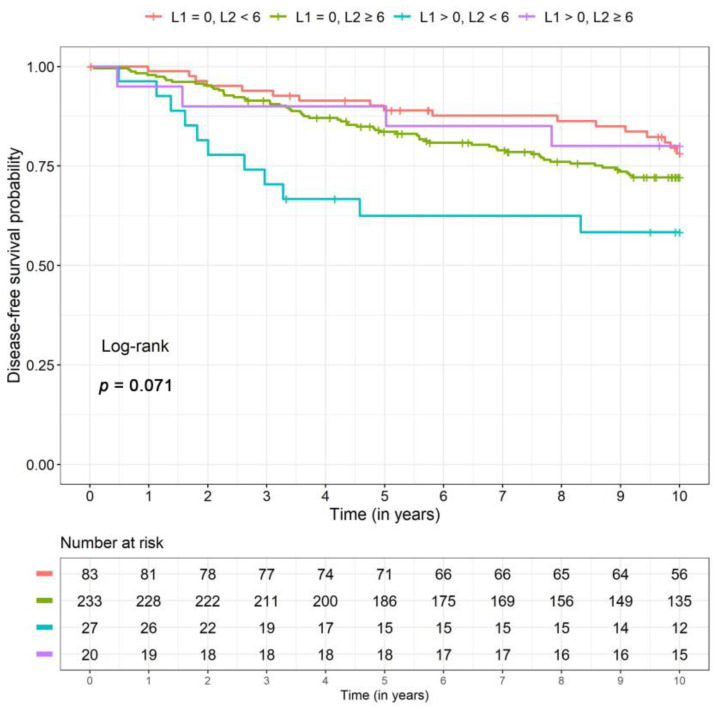
Kaplan–Meier curves for the four different combinations of PIWI-like 1 IRS (L1) and PIWI-like 2 IRS (L2) for disease-free survival.

**Figure 6:**
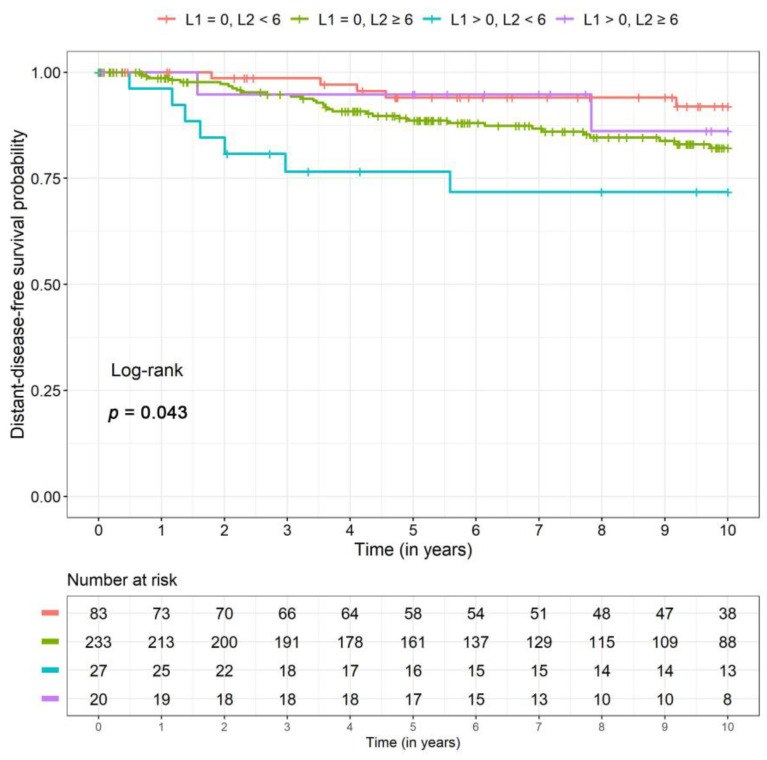
Kaplan–Meier curves for the four different combinations of PIWI-like 1 IRS (L1) and PIWI-like 2 IRS (L2) for distant disease-free survival.

For disease-free survival *(p =* 0.01) and distant disease-free survival *(p =* 0.003) the same interaction effect was recognizable. The patient group with high PIWI-like 1 IRS (>0) and low PIWI-like 2 IRS (<6) showed a significantly increased risk of DFS and DDFS (3.3- and 7.6-fold, respectively) compared to patients with low values for both parameters (Table 2). Again, DFS and DDFS survival rates were lower for this group with high PIWI-like 1 but low PIWI-like 2 expression. Note that event numbers were very small (Table 3). Kaplan–Meier curves for PIWI-like 1 IRS and PIWI-like 2 IRS can be found in Figure 5 and Figure 6, respectively.

### 2.5. Associations between PIWI-Like 1-IRS and Breast Cancer Subclasses

**Figure 7:**
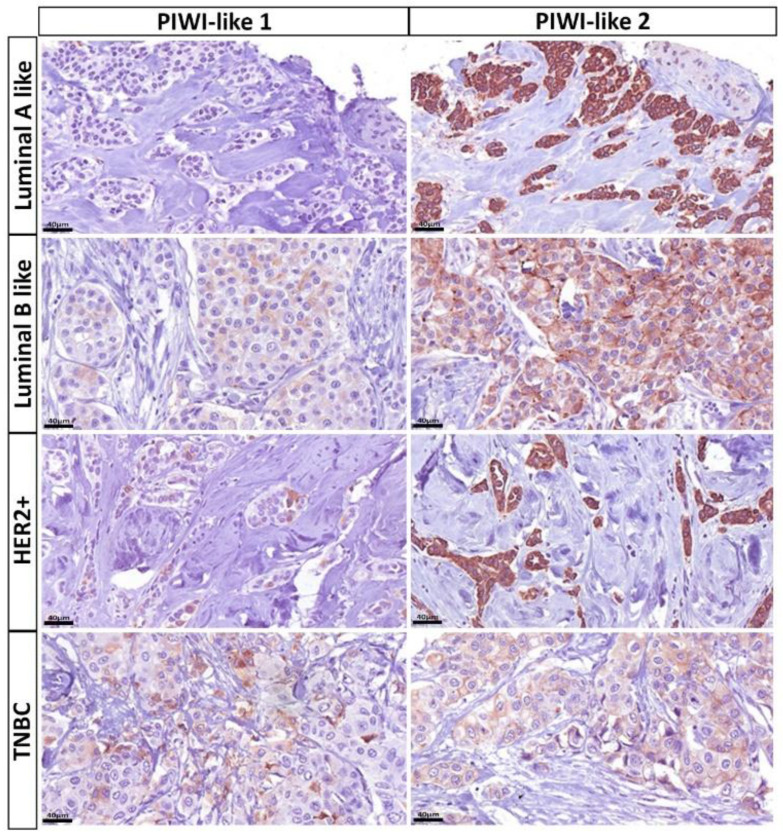
Immunohistochemical (IHC) expression patterns of PIWI-like 1 and PIWI-like 2 varied within the molecular-like (IHC) subtypes: tumors presented typically without PIWI-like 1 (IRS 0/12), but with strong and homogenous PIWI-like 2 expression (IRS 12/12). In contrast, Luminal B-like and HER2+ breast cancer cases depicted a slight expression of PIWI-like 1 in some tumor cells (IRS 2/12 and 4/12, respectively) with moderate or strong PIWI-like 2 staining (IRS 6/12 and 12/12, respectively). Triple-negative breast cancer (TNBC) showed increased PIWI-like 1 (IRS 4/12) and decreased PIWI-like 2 expression (IRS 3/12) when compared with Luminal A-like tumors.

**Figure 8:**
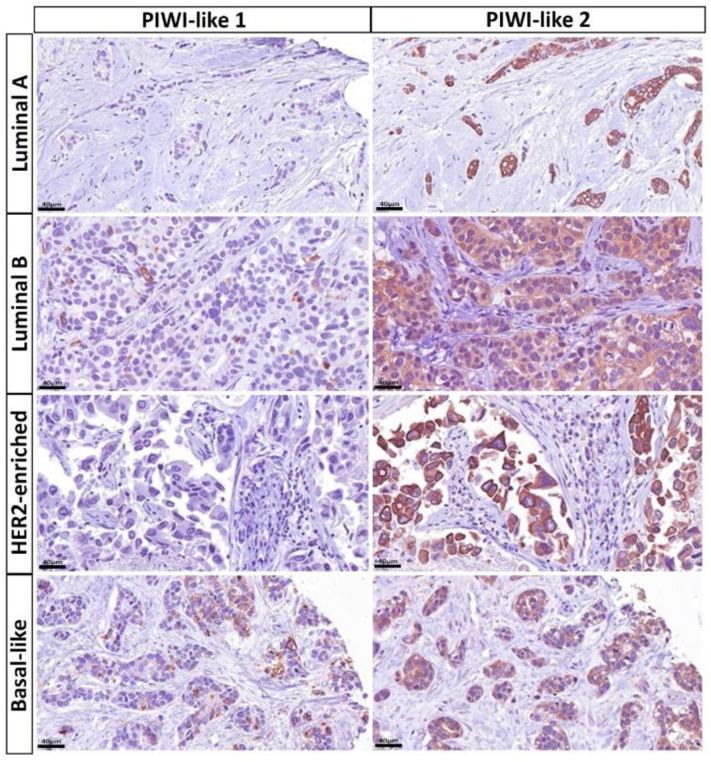
Immunohistochemical (IHC) expression patterns of PIWI-like 1 and PIWI-like 2 varied within the molecular PAM50 breast cancer subtypes: Luminal A breast cancer showed strong and homogenous PIWI-like 2 expression (IRS 12/12) in most cases, whereas there was no detection of PIWI-like 1 expression (IRS 0/12). In contrast, Luminal B and HER2-enriched tumors presented with either no or slight PIWI-like 1 expression (IRS 2/12 and 0/12, respectively). However, PIWI-like 2 staining was typically moderate to high (IRS 8/12 and 9/12, respectively). Basal-like breast cancer depicted increased PIWI-like 1 (IRS 4/12), but reduced PIWI-like 2 expression (IRS 6/12) when compared with Luminal A tumors.

For both molecular-like as well as PAM50 subtypes, PIWI-like 1 expression differed significantly between subgroups (both *p <* 0.001). With regard to molecular-like subtypes, breast cancers with no PIWI-like 1 expression (IRS = 0) exhibited higher proportions of the Luminal A like subtype (43.3%) followed by Luminal B like subtype (36.1%), whereas tumors with a PIWI-like 1 IRS expression greater than zero were most often found to exhibit Luminal B like subtype (48.9%) and triple-negative breast cancer (TNBC; 25.5%). Regarding the PAM50 subtype, 63.6% of patients with no PIWI-like 1 expression were assigned to Luminal A breast cancer, whereas most patients (38.3%) with PIWI-like 1 expressing tumors were allotted to basal-like, followed by the Luminal B subtype (25.5%). The HER2-enriched subtype was approximately three times more common in patients with PIWI-like 1 IRS > 0 compared to IRS = 0 (Table 4). Figure 7 and Figure 8 illustrate the typical expression patterns of PIWI-like 1 within the molecular-like (IHC) subtypes and the molecular PAM50 subtypes, respectively.

### 2.6. Associations between PIWI-Like 2 IRS and Breast Cancer Subclasses

Tumors with low PIWI-like 2 IRS (<6) were about six times more often assigned to TNBC (27.3%) and approximately half as often to the Luminal A like subtype (24.5%) compared to patients with high expression (IRS ≥ 6) (4.3% and 45.8%, respectively). Regarding the PAM50 subtype, patients with low PIWI-like-2-expressing cancer suffered about five times more often from basal-like (34.5%) and half as often from the Luminal A subtype (30.9%) compared to a high PIWI-like 2 expression (7.1% and 68.8%, respectively). The distributions of molecular-like and PAM50 subtypes were significantly different for patients with low and high expressions of PIWI-like 2 *(p <* 0.001, Table 4). The expression patterns of PIWI-like 2 within the molecular-like (IHC) subtypes and the molecular PAM50 subtypes are shown in Figure 7 and Figure 8, respectively.

## 3. Discussion

In this study, we investigated the protein expression of the PIWI proteins PIWI-like 1 and PIWI-like 2 by IHC in molecular breast cancer subtypes and their impact on survival of breast cancer patients.

The interaction of PIWI-like 1 and PIWI-like 2 was significantly associated with the survival of breast cancer patients. Hazard ratios, adjusted for known risk factors, significantly increased for OS (primary end point), as well as DFS and DDFS (secondary end points), when the tumor showed high PIWI-like 1 and low PIWI-like 2 expression compared to patients with other PIWI-like 1 and PIWI-like 2 combinations. Hence, the combination of PIWI-like 1 and PIWI-like 2 may be useful for risk stratification.

Both proteins showed a significant association with molecular (-like) subgroups. Whereas PIWI-like 1-negative tumors were more likely to be of the Luminal A (like) subtype, breast cancer with PIWI-like 1 expression was more often assigned to Luminal B (like) and TNBC subtypes—tumors that are known to behave more aggressive than Luminal A (like) tumors [22,23,24]. As for PIWI-like 1, significant association with molecular (-like) subtypes was again observed for PIWI-like 2. Patients with high expressions of this marker were predominantly seen in prognostic well-behaving Luminal A (like) tumors. Patients with lower PIWI-like 2 IRS values (<6) were more often assigned to Luminal B like and TNBC subtype. Hence, high PIWI-like 1 and low PIWI-like 2 are associated with more aggressive breast cancer subtypes, which is in line with the impact of these combined markers on survival.

In former studies, PIWI-like 1 and PIWI-like 2 were already shown to harbor prognostic value in a variety of tumor entities. PIWI-like 2 expression has been associated with prognostic impact on disease-specific survival and progressive-free survival in bladder cancer patients treated with chemotherapy [25]. In another cohort of muscle-invasive bladder cancer, prognostic effect was confirmed with PIWI-like 1 and PIWI-like 2 both being associated with disease-specific survival. PIWI-like 2 correlated with recurrence-free survival. Both proteins showed prognostic significance in bladder cancer subtypes [26]. PIWI-like 1 protein expression correlated with decreased cancer-specific survival, grading, tumor stage and distant metastases in renal cell carcinoma [27]. In soft tissue sarcoma, low PIWIL2 mRNA expression correlated significantly with poor prognosis [28].

In breast cancer, PIWIL1 and PIWIL3 gene expressions were reported to be upregulated, whereas PIWIL2 and PIWIL4 were downregulated compared with normal breast tissue. Although PIWIL3 and PIWIL4 were prognostic, PIWIL1 and PIWIL2 did not show significant impacts on survival [19]. In our study, however, PIWI-like 1 and PIWI-like 2 protein expression had significant prognostic effects regarding OS, DFS, and DDFS, and they were associated with molecular (-like) breast cancer subtypes. In one prior breast cancer study, the protein expressions of both markers were assessed in 101 invasive breast cancer cases and 31 mastopathy specimens. Corresponding to our findings, both markers showed cytoplasmic staining. Using a cut-off of IRS > 8, 26% and 11% cases were PIWI-like-1- and PIWI-like-2-positive, respectively. Expression of both proteins correlated significantly with each other and grading. Expressions of both were reported to be higher in breast cancer when compared to mastopathy. However, there was no significant prognostic effect regarding overall survival. Measuring the mRNA levels, PIWIL1 mRNA was not detected in non-neoplastic breast parenchyma, whereas PIWIL2 showed lower mRNA expression levels in breast cancer and mastopathy compared to normal breast tissue [21]. Another research group investigated the PIWI-like 2 expression in invasive and metastatic breast cancer using IHC and found expression of the assessed protein as either nuclear or cytoplasmic staining or both; in contrast to our investigation, only staining percentage was considered. Positive-stained nuclei correlated with Ki67 expression, whereas cytoplasmic positivity was associated with estrogen receptor (ER) expression [29]. Cao et al. analyzed PIWI-like 1 and PIWI-like 2 expressions in normal breast tissue, benign breast changes, and malignant breast cancer, and reported the highest expression levels in breast cancer with the lowest levels in normal breast parenchyma. Whereas PIWI-like 2 was not, PIWI-like 1 was a prognostic marker regarding survival. It was shown that poor outcomes resulted from down- and upregulation of transforming growth factor-β receptors and cyclin-dependent kinases CDK4, CDK6, and CDK8, respectively [30]. In one study, the breast cancer cell lines MCF-7, ZR-75.1, and SKBR3 BC cells and in mammary epithelial MCF10A cells were analyzed regarding PIWI expression. Whereas PIWI-like 2 and PIWI-like 4 were expressed, PIWI-like 1 and PIWI-like 3 were not detectable [7]. PIWI-like 2 was reported to be highly expressed in the TNBC cell line MDA-MB-231 in contrast to lower expression levels in the luminal breast cancer cell line MCF-7. The research group performed a Kaplan–Meier analysis based on data from an online database and found no significant prognostic effect of PIWIL1 and PIWIL2 regarding survival [31]. In a comprehensive breast cancer cohort, 31% of cases showed high PIWI-like 2 expression. This marker correlated with age, tumor size, histological type, tumor stage, and lymph node metastasis [32].

Thus far, we have not investigated piRNA expression in breast cancer. Multiple piRNAs have been described in breast cancer [19] with piRNA-4987, piRNA-20365, piRNA-20485, and piRNA-20582 being significantly overexpressed and piRNA-4987 correlating with lymph node metastases [33]. piRNA-36712 may hamper breast cancer progression and chemo-resistance harboring synergistic anti-tumor effects with taxane- and doxorubicin-based chemotherapy [34].

PIWI-like proteins can play a functional role in regulation of gene expression and chemotherapy response. PIWI-like 1 (Hiwi) is associated with global DNA hypermethylation and it translationally upregulates DNA methyltransferases (DNMT1 and DNMT3a) [35]. PIWI-like 2 mediates DNA repair through relaxation of chromatin by histone H3 acetylation [36]. In a mouse model, Mili (PIWIL2) modulates chromatin modifications upon cisplatin treatment and a decrease of H3 acetylation and a higher sensitivity to cisplatin in Mili-knockout mice embryonic fibroblasts has been reported [37]. In this way, PIWI-like protein expression may predict and their modification may affect therapy response to chemo- and DNA-/histone modification therapies.

One limitation of our study is that we studied the expression of PIWI-like 1 and PIWI-like 2 only using immunohistochemistry and we did not evaluate the mRNA level. However, immunohistochemical staining is an affordable method that can be easily implemented in routine diagnostics after an IHC biomarker has been proven to harbor any diagnostic, prognostic, or predictive impact. Nevertheless, investigating the relationship between PIWIL1 and PIWIL2 mRNA expression levels and PIWI-like 1 and PIWI-like 2 protein expressions can provide more insight into the regulation of the post-transcriptional/translational process for PIWI-like proteins. Another disadvantage of our study is that event numbers were very small. This led to a low power of tests. The survival analysis in the different molecular (-like) subgroups was limited due to a too-small number of cases. We were not able to correlate PIWI-like 1 and PIWI-like 2 expressions with pathologic complete response since most patients of our cohort were treated before the era of neoadjuvant therapy. Another limitation is that we did not investigate the relationship between PIWI-like 1/PIWI-like 2 and tumor progress/metastasizing and drug response/resistance. Further studies are needed to investigate these important issues. The strength of our investigation in comparison to former studies is that we analyzed a larger cohort of breast cancer cases and correlated our findings with molecular (-like) breast cancer subtypes and patients’ survival outcome.

## 4. Materials and Methods

### 4.1. Patient Selection

The Bavarian Breast Cancer Cases and Controls (BBCC) study represents a case–control study for investigation of molecular and epidemiological breast cancer risk, and prognostic and predictive parameters of the University Breast Center for Franconia (UBF, Bavaria, Germany) [38,39,40]. Eligibility of patients was constituted by the following criteria: Patients were at least 18 years of age and had been diagnosed with invasive breast cancer. Tissue of primary tumors was available in 894 of these patients with first diagnosis from 1997 to 2007 for the construction of a tissue microarray (TMA). For further analysis, patient groups (two male patients and 51 female patients with bilateral breast cancer at initial diagnosis, 50 metastases at initial diagnosis, and two with insufficient survival time) and cases without assessable PIWI-like 1/PIWI-like 2 status (426) were excluded (Figure 1). The final sample size contained 363 cases to analyze the influence of immunohistochemical (IHC) expression of PIWI-like 1 and PIWI-like 2 on outcome (Figure 1). All participants gave their informed consent for inclusion before they participated in the study. The study was conducted in accordance with the Declaration of Helsinki, and the protocol was approved by the Ethics Committee of the Medical Faculty of Erlangen University Hospital (ref. numbers 2700 and 297_17 Bc). There was no use of animal research within this project.

### 4.2. Clinical Data

Data collection has been reported in detail elsewhere [41,42]. Briefly, all clinical and histopathological data were compiled prospectively in an annually audited, certified database. Moreover, treatment procedures are audited annually as well, requiring treatment in accordance with the German guidelines for more than 95% of the patients [43,44]. Follow-up information regarding local recurrences, distant metastases, and death must be kept on file for a minimum of 10 years after the initial diagnosis.

### 4.3. Histopathological Assessment

Data on histological tumor type, tumor grading, estrogen receptor (ER), progesterone receptor (PR), and HER2 status were obtained from the original routine pathology reports. Grading was assessed on the pre-therapeutic formalin-fixed paraffin-embedded (FFPE) breast cancer core biopsies according to Elston and Ellis [45].

### 4.4. Immunohistochemical Staining of ER, PR, HER2, and Ki-67 and Molecular (-Like) Subtyping

IHC was conducted on the FFPE tissue of preoperative core biopsies according to routine standards of our institute and manufacturer’s instruction manual. For assessment of ER, PR, and Ki-67 IHC status, monoclonal mouse antibodies against ER-alpha (clone 1D5, 1:200 dilution, DAKO, Glostrup, Denmark), monoclonal mouse antibody against PgR (clone pgR636, 1:200 dilution, DAKO), and monoclonal antibody against Ki-67 (clone MIB-1, 1:200 dilution, DAKO) were used. The continuous percentage of positively-stained tumor cells was stated in the pathology reports; positive staining for ER and PR was time-dependently defined as ≥10% and ≥1%, respectively [46,47,48,49]. The cutoff for the proliferation marker Ki-67 was defined as 14% [50].

For HER2 IHC, a polyclonal antibody against HER2 (1:200 dilution, DAKO) was used, and the HER2 IHC score was documented in the pathology reports as 0, 1+, 2+, or 3+ in accordance with the published guidelines [51]. Tumors with a score of 0 or 1+ were considered HER2-negative and cases with a score of 3+ were defined as HER2-positive. Breast cancer samples with a 2+ staining were analyzed for gene copy numbers of *HER2* using chromogenic in situ hybridization (CISH). The *HER2* gene copy numbers (GCN) and the centromere GCN of the corresponding chromosome 17 were visualized using a kit with two probes of different colors (ZytoDot^®^ 2C SPEC *ERBB2*/*CEN17* Probe, ZytoVision GmbH, Bremerhaven, Germany). A case was regarded as *HER2* amplified if the *HER2*/*CEN17* ratio was ≥2.2 [52]. Before 2002, patients were retrospectively identified as being HER2-positive or -negative.

Definition of molecular-like subtypes was reported in detail earlier [53]. If the tumor had an HER2 IHC score of 3+ or showed an amplification of the *HER2* gene, HER2 status was considered positive (HER2-positive/HER2+ breast cancer) [54]. Cases with negative ER, PR, and HER2 status were defined as triple-negative breast cancer (TNBC). HER2-negative breast cancer with expression of either ER or PR were further separated into Luminal A-like (grading of 1 or 2) and Luminal B-like (grading of 3) tumors. PAM50 subtype (Luminal A, Luminal B, HER2-enriched, basal-like) was analyzed using the mRNA-based PAM50 gene expression analysis using the nCounter^®^ platform (NanoString Technologies, Seattle, WA, USA) [55].

### 4.5. Construction of Tissue Microarrays (BBCC TMA)

From the available FFPE blocks, areas containing invasive carcinoma of the breast were marked on a hematoxylin and eosin (H and E)-stained slide by an experienced pathologist. TMAs were created by re-embedding of cylindrical central breast cancer tissue core biopsies (1.0 mm per dot, tumor center) of several sample donor blocks into a single microarray block at defined coordinates.

### 4.6. PIWI-Like 1 and PIWI-Like 2 Immunohistochemistry

As described previously [25,27], a manual immunohistochemistry (PIWI-like 1/PIWI-like 2 IHC) protocol had been established for the evaluation of the PIWI-like 1 and PIWI-like 2 protein expression. For PIWI-like 1 IHC, a primary antibody against PIWI-like 1 (polyclonal goat IgG, N-17; Cat. No. 22685; dilution 1:50; Santa Cruz, Heidelberg, Germany), and for PIWI-like 2 IHC, a primary antibody against PIWI-like 2 (polyclonal goat IgG, K-18; cat. no. sc67502; dilution 1:50; Santa Cruz, Heidelberg, Germany) was applied for 30 min after heat pretreatment at 120 °C for 5 min with tris-ethylenediamine tetraacetic acid (EDTA) buffer, pH 9, and peroxidase blocking (DAKO, Hamburg, Germany). Incubation with a horseradish peroxidase (HRP)-labeled secondary antibody polymer (EnVision, DAKO, Hamburg, Germany) was conducted for 30 min followed by adding a diaminobenzidine (DAB) substrate chromogen solution (DAKO, Hamburg, Germany) for 10 min and counterstaining for 1 min with hematoxylin (Merck, Darmstadt, Germany). Incubation procedures were performed at room temperature. Positive controls, as well as negative control slides without the addition of primary antibody, were included for each staining experiment.

The stained TMA slides were evaluated with a Zeiss Axio Imager A1 microscope (magnification of 100× and 200×) (Carl Zeiss, Jena, Germany) by a pathologist specialized on breast cancer (R.E.). Since nuclear staining was missing in almost all cases, only cytoplasmic staining was evaluated per core. Both, percentage (0–100%, categorial: 0% = 0, <10% = 1, 10–50% = 2, 51–80% = 3, and >80% = 4) and intensity (no staining = 0; weak = 1, moderate = 2, and strong = 3) of stained tumor cells and nonneoplastic cells were analyzed. Multiplying intensity and categorical percentage, we received an immunoreactive score (IRS) from 0/12 to 12/12 (0–12, excluding the values 5, 7, 10, and 11) according to Remmele and Stegner [56].

### 4.7. Statistical Analysis

The primary objective was to investigate whether the biomarkers PIWI-like 1 and PIWI-like 2 had prognostic value on overall survival (OS) in addition to well-known prognostic patient and tumor characteristics. Similar analyses for disease-free survival (DFS) and distant-disease-free survival (DDFS) were secondary objectives.

OS was defined as the time from the date of primary diagnosis to the earliest date of death from any cause or the date of censoring. Patients who were lost to follow-up before the maximal observation time of 10 years or were alive after the maximal observation time were censored at the last date they were known to be alive or at the maximum observation time. DFS was defined in a similar fashion, including the events of distant metastasis and local recurrence. For DDFS only events of distant metastasis were counted.

A multivariable Cox regression model was fitted with OS, DFS, and DDFS, separately, as outcome and the following predictors: age at diagnosis (continuous), tumor stage (ordinal, T1 to T4), lymph node status (categorical; N0, N+), molecular-like class (categorical; TNBC, Luminal A like, Luminal B like, HER2+), PIWI-like 1 IRS, PIWI-like 2 IRS, and the interaction of PIWI-like 1 IRS and PIWI-like 2 IRS. Both IRS variables entered the model’s binary: For PIWI-like 1 IRS, the chosen cutoff was 0, as most observations (87.1%) were 0. For PIWI-like 2 IRS the median (=6) of all observations was used as cutoff. The proportional hazards assumptions were checked using the method of Grambsch and Therneau [57]. Survival rates were estimated using the Kaplan–Meier product limit method.

When the interaction of PIWI-like 1 and PIWI-like 2 was significant in the multivariable Cox model, hazard ratios (HR) were calculated for patient subgroups defined by PIWI-like 1 and PIWI-like 2, using the interaction model. In case of non-significance, interactions were removed from the model and HRs from this reduced model were extracted for PIWI-like 1 and PIWI-like 2.

A further secondary objective was to examine associations between PIWI-like 1/PIWI-like 2 and molecular-like subtype as well as PAM50 subtype. Therefore, chi-squared tests were used. Missing values for the variable PAM50 subtype were imputed using multinomial regression (with Ki-67 and molecular-like subtype as regressors). Other variables, where the proportion of missing values was small (<10%), were replaced as done in Salmen et al. [58]. Subjects with missing survival information and missing values in the biomarker of interest (PIWI-like 1/PIWI-like 2) were excluded from analysis.

All of the tests were two-sided, and *p <* 0.05 was regarded as statistically significant. Analyses were carried out using the R system for statistical computing (version 3.6.1; R Development Core Team, Vienna, Austria, 2019).

## 5. Conclusions

In invasive breast cancer, the interaction of PIWI-like 1 and PIWI-like 2 protein expression was significantly associated with patients’ outcome (OS, DFS, and DDFS) and both markers were significantly associated with molecular subtyping. The combination of high PIWI-like 1 and low PIWI-like 2 expression was associated with poorer prognosis and more aggressive breast cancer subtypes. Further studies are needed to confirm our findings and to gain more insight into the influence and molecular mechanism of PIWI-like 1 and PIWI-like 2 on breast cancer.

## Figures and Tables

**Table 1:** Baseline characteristics of clinical and pathologic parameters for the tumor center tissue-dataset.

Parameter		*n* (%) or Median (IQR)
*n*		363 (100%)
Age (years)		58 (49, 67)
BMI (kg/m^2^)		25.7 (23.1, 28.6)
Ki-67 (%)		15 (10, 30)
ER	Positive	298 (82.1%)
PR	Positive	276 (76.0%)
HER2	Positive	42 (11.6%)
Grading (G)	G1	30 (8.3%)
	G2	248 (68.3%)
	G3	85 (23.4%)
Nodal status (N)	N+	148 (40.8%)
Tumor stage (T)	T1	186 (51.2%)
	T2	138 (38.0%)
	T3	19 (5.2%)
	T4	20 (5.5%)
Molecular-like subtype	TNBC	41 (11.3%)
	Luminal A like	143 (39.4%)
	Luminal B like	137 (37.7%)
	HER2+	42 (11.6%)
PAM50 subtype	Basal-like	56 (15.4%)
	Luminal A	208 (57.3%)
	Luminal B	69 (19.0%)
	HER2-enriched	30 (8.3%)
PIWI-like 1 IRS	Median	0 (0, 0)
	IRS > 0	47 (12.9%)
PIWI-like 2 IRS	Median	6 (4, 8)
	IRS ≥ 6	253 (69.7%)

Abbreviations: BMI—body mass index; ER—estrogen receptor; HER2—human epidermal growth factor receptor 2; IQR—interquartile range; IRS—immunoreactive score; PR—progesterone receptor; TNBC—triple-negative breast cancer.

**Table 2:** Adjusted and unadjusted hazard ratios (HR) and 95% confidence intervals (CIs) for the four combinations of PIWI-Like 1 IRS and PIWI-Like 2 IRS.

Combination of PIWI-Like 1 IRS (L1) and PIWI-Like 2 IRS (L2)		Adjusted		Unadjusted	
		HR (95% CI)	*p*-Value	HR (95% CI)	*p*-Value
OS 79 events	L1 = 0, L2 < 6	Reference	–	Reference	–
	L1 = 0, L2 ≥ 6	1.36 (0.75, 2.46)	0.31	1.30 (0.73, 2.31)	0.37
	L1 > 0, L2 < 6	2.92 (1.24, 6.90)	0.01	2.26 (0.99, 5.17)	0.05
	L1 > 0, L2 ≥ 6	0.89 (0.29, 2.69)	0.83	1.08 (0.36, 3.27)	0.89
DFS 94 events	L1 = 0, L2 < 6	Reference	–	Reference	–
	L1 = 0, L2 ≥ 6	1.34 (0.77, 2.31)	0.30	1.38 (0.81, 2.36)	0.24
	L1 > 0, L2 < 6	3.27 (1.48, 7.20)	0.003	2.61 (1.22, 5.59)	0.01
	L1 > 0, L2 ≥ 6	0.83 (0.28, 2.48)	0.74	0.97 (0.33, 2.88)	0.96
DDFS 46 events	L1 = 0, L2 < 6	Reference	–	Reference	–
	L1 = 0, L2 ≥ 6	2.73 (1.04, 7.18)	0.04	2.33 (0.91, 5.98)	0.08
	L1 > 0, L2 < 6	7.64 (2.35, 24.82)	<0.001	4.72 (1.50, 14.88)	0.008
	L1 > 0, L2 ≥ 6	1.19 (0.23, 6.24)	0.83	1.52 (0.29, 7.83)	0.62

Note: HRs adjusted for age at diagnosis, tumor stage, lymph node status, molecular-like class. Abbreviations: 95% CI—95% confidence interval; DFS—disease-free survival; DDFS—distant disease-free survival; HR—hazard ratio; IRS—immunoreactive score; L1—PIWI-like 1 IRS; L2—PIWI-like 2 IRS; OS—overall survival.

**Table 3:** Survival rates (5 and 10 years) including 95% confidence intervals for the four combinations of PIWI-like 1 and PIWI-like 2 resulting from the unadjusted model (corresponding to Figure 4, Figure 5 and Figure 6).

Combination of PIWI-Like 1 IRS (L1) and PIWI-Like 2 IRS (L2)		L1 = 0, L2 < 6	L1 = 0, L2 ≥ 6	L1 > 0, L2 < 6	L1 > 0, L2 ≥ 6
Total	*n*	83	233	27	20
OS	n events	15	51	9	4
	5-year SR (CI)	0.91 (0.87, 0.96)	0.89 (0.85, 0.92)	0.81 (0.70, 0.94)	0.90 (0.82, 1.00)
	10-year SR (CI)	0.81 (0.73, 0.90)	0.77 (0.71, 0.82)	0.63 (0.46, 0.85)	0.80 (0.64, 1.00)
DFS	n events	17	62	11	4
	5-year SR (CI)	0.88 (0.82, 0.94)	0.83 (0.79, 0.88)	0.71 (0.57, 0.87)	0.88 (0.77, 1.00)
	10-year SR (CI)	0.79 (0.71, 0.88)	0.72 (0.66, 0.78)	0.54 (0.37, 0.78)	0.80 (0.64, 1.00)
DDFS	n events	5	32	7	2
	5-year SR (CI)	0.95 (0.90, 0.99)	0.88 (0.84, 0.93)	0.78 (0.64, 0.94)	0.92 (0.82, 1.00)
	10-year SR (CI)	0.92 (0.86, 0.99)	0.83 (0.77, 0.88)	0.68 (0.51, 0.90)	0.88 (0.74, 1.00)

Abbreviations: CI—95% confidence interval; DFS—disease-free survival; DDFS—distant disease-free survival; L1—PIWI-like 1 IRS; L2—PIWI-like 2 IRS; OS—overall survival; SR—survival rate.

**Table 4:** Distribution (*n* and %) of PIWI-like 1 IRS and PIWI-like 2 IRS by molecular-like subclasses and PAM50 subtypes including the *p* value of the chi-squared test.

**Parameter**		**PIWI-Like 1 IRS = 0**	**PIWI-Like 1 IRS > 0**	***p*-Value**
Molecular-like subtype	TNBC	29 (9.2%)	12 (25.5%)	<0.001
	Luminal A like	137 (43.3%)	6 (12.8%)	
	Luminal B like	114 (36.1%)	23 (48.9%)	
	HER2+	36(11.4%)	6 (12.8%)	
PAM50 subtype	Basal-like	38 (12.0%)	18 (38.3%)	<0.001
	Luminal A	201 (63.6%)	7 (14.9%)	
	Luminal B	57 (18.0%)	12 (25.5%)	
	HER2-enriched	20 (6.3%)	10 (21.3%)	
**Parameter**		**PIWI-Like 2 IRS < 6**	**PIWI-Like 2 IRS ≥ 6**	***p*-Value**
Molecular-like subtype	TNBC	30 (27.3%)	11 (4.3%)	<0.001
	Luminal A like	27 (24.5%)	116 (45.8%)	
	Luminal B like	42 (38.2%)	95 (37.5%)	
	HER2+	11 (10.0%)	31 (12.3%)	
PAM50 subtype	Basal-like	38 (34.5%)	18 (7.1%)	<0.001
	Luminal A	34 (30.9%)	174 (68.8%)	
	Luminal B	26 (23.6%)	43 (17.0%)	
	HER2-enriched	12 (10.9%)	18 (7.1%)	

Abbreviations: HER2—human epidermal growth factor receptor 2; IRS—immunoreactive score; TNBC—triple-negative breast cancer.

## References

[1] Sasaki T., Shiohama A., Minoshima S., Shimizu N. (2003). Identification of eight members of the Argonaute family in the human genome. Genomics.

[2] Litwin M., Szczepańska-Buda A., Piotrowska A., Dzięgiel P., Witkiewicz W. (2017). The meaning of PIWI proteins in cancer development. Oncol. Lett..

[3] Livak K.J. (1990). Detailed structure of the Drosophila melanogaster stellate genes and their transcripts. Genetics.

[4] Cox D.N., Chao A., Baker J., Chang L., Qiao D., Lin H. (1998). A novel class of evolutionarily conserved genes defined by piwi are essential for stem cell self-renewal. Genes Dev..

[5] Ishizu H., Siomi H., Siomi M.C. (2012). Biology of PIWI-interacting RNAs: New insights into biogenesis and function inside and outside of germlines. Genes Dev..

[6] Girard A., Sachidanandam R., Hannon G.J., Carmell M.A. (2006). A germline-specific class of small RNAs binds mammalian Piwi proteins. Nature.

[7] Hashim A., Rizzo F., Marchese G., Ravo M., Tarallo R., Nassa G., Giurato G., Santamaria G., Cordella A., Cantarella C. (2014). RNA sequencing identifies specific PIWI-interacting small non-coding RNA expression patterns in breast cancer. Oncotarget.

[8] Ye Y., Yin D.T., Chen L., Zhou Q., Shen R., He G., Yan Q., Tong Z., Issekutz A.C., Shapiro C.L. (2010). Identification of Piwil2-like (PL2L) proteins that promote tumorigenesis. PLoS ONE.

[9] Al-Janabi O., Wach S., Nolte E., Weigelt K., Rau T.T., Stohr C., Legal W., Schick S., Greither T., Hartmann A. (2014). Piwi-like 1 and 4 gene transcript levels are associated with clinicopathological parameters in renal cell carcinomas. Biochim. Biophys. Acta.

[10] Suzuki R., Honda S., Kirino Y. (2012). PIWI Expression and Function in Cancer. Front. Genet..

[11] Tan Y., Liu L., Liao M., Zhang C., Hu S., Zou M., Gu M., Li X. (2015). Emerging roles for PIWI proteins in cancer. Acta Biochim. Biophys. Sin..

[12] Fathizadeh H., Asemi Z. (2019). Epigenetic roles of PIWI proteins and piRNAs in lung cancer. Cell Biosci..

[13] Zeng Y., Qu L.K., Meng L., Liu C.Y., Dong B., Xing X.F., Wu J., Shou C.C. (2011). HIWI expression profile in cancer cells and its prognostic value for patients with colorectal cancer. Chin. Med. J..

[14] Zhao Y.M., Zhou J.M., Wang L.R., He H.W., Wang X.L., Tao Z.H., Sun H.C., Wu W.Z., Fan J., Tang Z.Y. (2012). HIWI is associated with prognosis in patients with hepatocellular carcinoma after curative resection. Cancer.

[15] Liu W., Gao Q., Chen K., Xue X., Li M., Chen Q., Zhu G., Gao Y. (2014). Hiwi facilitates chemoresistance as a cancer stem cell marker in cervical cancer. Oncol. Rep..

[16] Li L., Yu C., Gao H., Li Y. (2010). Argonaute proteins: Potential biomarkers for human colon cancer. BMC Cancer.

[17] Li D., Sun X., Yan D., Huang J., Luo Q., Tang H., Peng Z. (2012). Piwil2 modulates the proliferation and metastasis of colon cancer via regulation of matrix metallopeptidase 9 transcriptional activity. Exp. Biol. Med. (Maywood).

[18] Lee J.H., Schutte D., Wulf G., Fuzesi L., Radzun H.J., Schweyer S., Engel W., Nayernia K. (2006). Stem-cell protein Piwil2 is widely expressed in tumors and inhibits apoptosis through activation of Stat3/Bcl-XL pathway. Hum. Mol. Genet..

[19] Krishnan P., Ghosh S., Graham K., Mackey J.R., Kovalchuk O., Damaraju S. (2016). Piwi-interacting RNAs and PIWI genes as novel prognostic markers for breast cancer. Oncotarget.

[20] Wang D.W., Wang Z.H., Wang L.L., Song Y., Zhang G.Z. (2014). Overexpression of hiwi promotes growth of human breast cancer cells. Asian Pac. J. Cancer Prev. APJCP.

[21] Litwin M., Szczepanska-Buda A., Michalowska D., Grzegrzolka J., Piotrowska A., Gomulkiewicz A., Wojnar A., Dziegiel P., Witkiewicz W. (2018). Aberrant Expression of PIWIL1 and PIWIL2 and Their Clinical Significance in Ductal Breast Carcinoma. Anticancer Res..

[22] Perou C.M., Sorlie T., Eisen M.B., van de Rijn M., Jeffrey S.S., Rees C.A., Pollack J.R., Ross D.T., Johnsen H., Akslen L.A. (2000). Molecular portraits of human breast tumours. Nature.

[23] Sorlie T., Perou C.M., Tibshirani R., Aas T., Geisler S., Johnsen H., Hastie T., Eisen M.B., van de Rijn M., Jeffrey S.S. (2001). Gene expression patterns of breast carcinomas distinguish tumor subclasses with clinical implications. Proc. Natl Acad. Sci. USA.

[24] Parker J.S., Mullins M., Cheang M.C., Leung S., Voduc D., Vickery T., Davies S., Fauron C., He X., Hu Z. (2009). Supervised risk predictor of breast cancer based on intrinsic subtypes. J. Clin. Oncol..

[25] Taubert H., Wach S., Jung R., Pugia M., Keck B., Bertz S., Nolte E., Stoehr R., Lehmann J., Ohlmann C.-H. (2015). Piwil 2 Expression Is Correlated with Disease-Specific and Progression-Free Survival of Chemotherapy-Treated Bladder Cancer Patients. Mol. Med..

[26] Eckstein M., Jung R., Weigelt K., Sikic D., Stohr R., Geppert C., Agaimy A., Lieb V., Hartmann A., Wullich B. (2018). Piwi-like 1 and -2 protein expression levels are prognostic factors for muscle invasive urothelial bladder cancer patients. Sci. Rep..

[27] Stöhr C.G., Steffens S., Polifka I., Jung R., Kahlmeyer A., Ivanyi P., Weber F., Hartmann A., Wullich B., Wach S. (2019). Piwi-like 1 protein expression is a prognostic factor for renal cell carcinoma patients. Sci. Rep..

[28] Greither T., Koser F., Kappler M., Bache M., Lautenschläger C., Göbel S., Holzhausen H.-J., Wach S., Würl P., Taubert H. (2012). Expression of human Piwi-like genes is associated with prognosis for soft tissue sarcoma patients. BMC Cancer.

[29] Liu J.J., Shen R., Chen L., Ye Y., He G., Hua K., Jarjoura D., Nakano T., Ramesh G.K., Shapiro C.L. (2010). Piwil2 is expressed in various stages of breast cancers and has the potential to be used as a novel biomarker. Int. J. Clin. Exp. Pathol..

[30] Cao J., Xu G., Lan J., Huang Q., Tang Z., Tian L. (2016). High expression of piwi-like RNA-mediated gene silencing 1 is associated with poor prognosis via regulating transforming growth factor-beta receptors and cyclin-dependent kinases in breast cancer. Mol. Med. Rep..

[31] Wang Z., Liu N., Shi S., Liu S., Lin H. (2016). The Role of PIWIL4, an Argonaute Family Protein, in Breast Cancer. J. Biol. Chem..

[32] Zhang H., Ren Y., Xu H., Pang D., Duan C., Liu C. (2013). The expression of stem cell protein Piwil2 and piR-932 in breast cancer. Surg. Oncol..

[33] Huang G., Hu H., Xue X., Shen S., Gao E., Guo G., Shen X., Zhang X. (2013). Altered expression of piRNAs and their relation with clinicopathologic features of breast cancer. Clin. Transl. Oncol..

[34] Tan L., Mai D., Zhang B., Jiang X., Zhang J., Bai R., Ye Y., Li M., Pan L., Su J. (2019). PIWI-interacting RNA-36712 restrains breast cancer progression and chemoresistance by interaction with SEPW1 pseudogene SEPW1P RNA. Mol. Cancer.

[35] Siddiqi S., Terry M., Matushansky I. (2012). Hiwi mediated tumorigenesis is associated with DNA hypermethylation. PLoS ONE.

[36] Yin D.-T., Wang Q., Chen L., Liu M.-Y., Han C., Yan Q., Shen R., He G., Duan W., Li J.-J. (2011). Germline stem cell gene PIWIL2 mediates DNA repair through relaxation of chromatin. PLoS ONE.

[37] Wang Q.-E., Han C., Milum K., Wani A.A. (2011). Stem cell protein Piwil2 modulates chromatin modifications upon cisplatin treatment. Mutat. Res..

[38] Fasching P.A., Weihbrecht S., Haeberle L., Gasparyan A., Villalobos I.E., Ma Y., Ekici A.B., Wachter D.L., Hartmann A., Beckmann M.W. (2014). HER2 and TOP2A amplification in a hospital-based cohort of breast cancer patients: Associations with patient and tumor characteristics. Breast Cancer Res. Treat..

[39] Serce N.B., Boesl A., Klaman I., von Serenyi S., Noetzel E., Press M.F., Dimmler A., Hartmann A., Sehouli J., Knuechel R. (2012). Overexpression of SERBP1 (Plasminogen activator inhibitor 1 RNA binding protein) in human breast cancer is correlated with favourable prognosis. BMC Cancer.

[40] Bektas N., Noetzel E., Veeck J., Press M.F., Kristiansen G., Naami A., Hartmann A., Dimmler A., Beckmann M.W., Knuchel R. (2008). The ubiquitin-like molecule interferon-stimulated gene 15 (ISG15) is a potential prognostic marker in human breast cancer. Breast Cancer Res..

[41] Brennan M., Gass P., Häberle L., Wang D., Hartmann A., Lux M.P., Beckmann M.W., Untch M., Fasching P.A. (2018). The effect of participation in neoadjuvant clinical trials on outcomes in patients with early breast cancer. Breast Cancer Res. Treat..

[42] Beckmann M.W., Brucker C., Hanf V., Rauh C., Bani M.R., Knob S., Petsch S., Schick S., Fasching P.A., Hartmann A. (2011). Quality assured health care in certified breast centers and improvement of the prognosis of breast cancer patients. Onkologie.

[43] Wockel A., Festl J., Stuber T., Brust K., Stangl S., Heuschmann P.U., Albert U.S., Budach W., Follmann M., Janni W. (2018). Interdisciplinary Screening, Diagnosis, Therapy and Follow-up of Breast Cancer. Guideline of the DGGG and the DKG (S3-Level, AWMF Registry Number 032/045OL, December 2017)—Part 1 with Recommendations for the Screening, Diagnosis and Therapy of Breast Cancer. Geburtshilfe Frauenheilkd..

[44] Wockel A., Festl J., Stuber T., Brust K., Krockenberger M., Heuschmann P.U., Jiru-Hillmann S., Albert U.S., Budach W., Follmann M. (2018). Interdisciplinary Screening, Diagnosis, Therapy and Follow-up of Breast Cancer. Guideline of the DGGG and the DKG (S3-Level, AWMF Registry Number 032/045OL, December 2017)—Part 2 with Recommendations for the Therapy of Primary, Recurrent and Advanced Breast Cancer. Geburtshilfe Frauenheilkd..

[45] Elston C.W., Ellis I.O. (1991). Pathological prognostic factors in breast cancer. I. The value of histological grade in breast cancer: Experience from a large study with long-term follow-up. Histopathology.

[46] Goldhirsch A., Wood W.C., Gelber R.D., Coates A.S., Thürlimann B., Senn H.-J. (2003). Meeting Highlights: Updated International Expert Consensus on the Primary Therapy of Early Breast Cancer. J. Clin. Oncol..

[47] Goldhirsch A., Glick J.H., Gelber R.D., Coates A.S., Thurlimann B., Senn H.J. (2005). Meeting highlights: International expert consensus on the primary therapy of early breast cancer 2005. Ann. Oncol. Off. J. Eur. Soc. Med. Oncol..

[48] Hammond M.E., Hayes D.F., Dowsett M., Allred D.C., Hagerty K.L., Badve S., Fitzgibbons P.L., Francis G., Goldstein N.S., Hayes M. (2010). American Society of Clinical Oncology/College of American Pathologists guideline recommendations for immunohistochemical testing of estrogen and progesterone receptors in breast cancer (unabridged version). Arch. Pathol. Lab. Med..

[49] Hammond M.E., Hayes D.F., Wolff A.C. (2011). Clinical Notice for American Society of Clinical Oncology-College of American Pathologists guideline recommendations on ER/PgR and HER2 testing in breast cancer. J. Clin. Oncol..

[50] Goldhirsch A., Glick J.H., Gelber R.D., Coates A.S., Senn H.J. (2001). Meeting highlights: International Consensus Panel on the Treatment of Primary Breast Cancer. Seventh International Conference on Adjuvant Therapy of Primary Breast Cancer. J. Clin. Oncol..

[51] Sauter G., Lee J., Bartlett J.M., Slamon D.J., Press M.F. (2009). Guidelines for human epidermal growth factor receptor 2 testing: Biologic and methodologic considerations. J. Clin. Oncol..

[52] Wolff A.C., Hammond M.E., Schwartz J.N., Hagerty K.L., Allred D.C., Cote R.J., Dowsett M., Fitzgibbons P.L., Hanna W.M., Langer A. (2007). American Society of Clinical Oncology/College of American Pathologists guideline recommendations for human epidermal growth factor receptor 2 testing in breast cancer. Arch. Pathol. Lab. Med..

[53] Wunderle M., Pretscher J., Brucker S.Y., Volz B., Hartmann A., Fiessler C., Hein A., Häberle L., Jud S.M., Lux M.P. (2019). Association between breast cancer risk factors and molecular type in postmenopausal patients with hormone receptor-positive early breast cancer. Breast Cancer Res. Treat..

[54] Bethune G.C., Veldhuijzen van Zanten D., MacIntosh R.F., Rayson D., Younis T., Thompson K., Barnes P.J. (2015). Impact of the 2013 American Society of Clinical Oncology/College of American Pathologists guideline recommendations for human epidermal growth factor receptor 2 (HER2) testing of invasive breast carcinoma: A focus on tumours assessed as ‘equivocal’ for HER2 gene amplification by fluorescence in-situ hybridization. Histopathology.

[55] Nielsen T., Wallden B., Schaper C., Ferree S., Liu S., Gao D., Barry G., Dowidar N., Maysuria M., Storhoff J. (2014). Analytical validation of the PAM50-based Prosigna Breast Cancer Prognostic Gene Signature Assay and nCounter Analysis System using formalin-fixed paraffin-embedded breast tumor specimens. BMC Cancer.

[56] Remmele W., Stegner H.E. (1987). Recommendation for uniform definition of an immunoreactive score (IRS) for immunohistochemical estrogen receptor detection (ER-ICA) in breast cancer tissue. Pathologe.

[57] Grambsch P.M., Therneau T.M. (1994). Proportional Hazards Tests and Diagnostics Based on Weighted Residuals. Biometrika.

[58] Salmen J., Neugebauer J., Fasching P.A., Haeberle L., Huober J., Wöckel A., Rauh C., Schuetz F., Weissenbacher T., Kost B. (2014). Pooled analysis of the prognostic relevance of progesterone receptor status in five German cohort studies. Breast Cancer Res. Treat..

